# An interferon-related signature characterizes the whole blood transcriptome profile of insulin-resistant individuals—the CODAM study

**DOI:** 10.1186/s12263-021-00702-7

**Published:** 2021-12-09

**Authors:** Marianthi Kalafati, Martina Kutmon, Chris T. Evelo, Carla J. H. van der Kallen, Casper G. Schalkwijk, Coen D. A. Stehouwer, B. I. O. S. Consortium, Ellen E. Blaak, Marleen M. J. van Greevenbroek, Michiel Adriaens

**Affiliations:** 1 Department of Human Biology, School of Nutrition and Translational Research in Metabolism (NUTRIM), Maastricht University, Maastricht, The Netherlands; 2 Department of Bioinformatics – BiGCaT, School of Nutrition and Translational Research in Metabolism (NUTRIM), Maastricht University, Maastricht, The Netherlands; 3 Maastricht Centre for Systems Biology (MaCSBio), Maastricht University, Maastricht, The Netherlands; 4 Department of Internal Medicine, School for Cardiovascular Diseases (CARIM), Maastricht University, Maastricht, The Netherlands

**Keywords:** Insulin resistance, Obesity, Whole blood transcriptome, Monocytes, Inter-individual differences, Interferon signature

## Abstract

**Background:**

Worldwide, the prevalence of obesity and insulin resistance has grown dramatically. Gene expression profiling in blood represents a powerful means to explore disease pathogenesis, but the potential impact of inter-individual differences in a cell-type profile is not always taken into account. The objective of this project was to investigate the whole blood transcriptome profile of insulin-resistant as compared to insulin-sensitive individuals independent of inter-individual differences in white blood cell profile.

**Results:**

We report a 3% higher relative amount of monocytes in the insulin-resistant individuals. Furthermore, independent of their white blood cell profile, insulin-resistant participants had (i) higher expression of interferon-stimulated genes and (ii) lower expression of genes involved in cellular differentiation and remodeling of the actin cytoskeleton.

**Conclusions:**

We present an approach to investigate the whole blood transcriptome of insulin-resistant individuals, independent of their DNA methylation-derived white blood cell profile. An interferon-related signature characterizes the whole blood transcriptome profile of the insulin-resistant individuals, independent of their white blood cell profile. The observed signature indicates increased systemic inflammation possibly due to an innate immune response and whole-body insulin resistance, which can be a cause or a consequence of insulin resistance. Altered gene expression in specific organs may be reflected in whole blood; hence, our results may reflect obesity and/or insulin resistance-related organ dysfunction in the insulin-resistant individuals.

**Supplementary Information:**

The online version contains supplementary material available at 10.1186/s12263-021-00702-7.

## Background

Obesity is one of the most important risk factors for insulin resistance, and it is linked to low-grade inflammation [1]. In general, in an obese adipose tissue, the state of low-grade inflammation has been predominantly attributed to the accumulation of pro-inflammatory macrophages and other immune cells that produce and secrete pro-inflammatory cytokines [2]. Studies performed in human and mouse have shown that accumulation of T cells precedes macrophage accumulation in obese adipose tissue [3] and that numerous changes in their subpopulations are also linked to the development of obesity and insulin resistance in animal models [4]. On the same note, an increased white blood count was associated with the development of insulin resistance [5]. Insulin resistance has been shown to positively correlate with the neutrophil-lymphocyte ratio [6] and plasma insulin concentration to positively correlate with the numbers of lymphocytes and monocytes [7].

The study of obesity-associated insulin resistance can be performed in metabolically active tissues, such as adipose tissue, liver. or muscle, using gene expression profiling. Gene expression profiling of whole blood [8] or PBMCs [9] is also considered for the study of insulin resistance. It has been shown that approximately 80% of the whole blood transcriptome gene expression was shared with any given tissue [10]. Additionally, the changes in the expression levels of individual genes reflect alterations in the environment of whole blood or blood cells and may also reflect organ-specific changes [10]. Hence, blood may be a beneficial tool for studying insulin resistance, diabetes, and obesity. However, differential gene expression analysis performed in the blood does not always take into account the potential impact from the inter-individual differences in a cell-type profile. These differences might represent changes on the cell type-specific level but also reflect changes in biologically relevant tissues.

Given a tissue, a cell-type profile can be determined experimentally, such as fluorescence-activated cell sorting, or computationally [11]. In this paper, we present a computational approach to investigate the whole blood transcriptome of individuals from the Cohort on Diabetes and Atherosclerosis Maastricht (CODAM) [12] study, a prospective observational cohort that includes participants with an elevated risk of type 2 diabetes mellitus (T2DM) and cardiovascular disease. In this approach, we used the computational algorithm EpiDISH [13] to infer the white blood cell (WBC) profile from whole blood DNA methylation data. Next, we analyzed RNA sequencing data in the whole blood of the same group of participants. The objective of this project was to investigate the whole blood transcriptome profile of insulin-resistant as compared to insulin-sensitive individuals independent of inter-individual differences in their WBC profile.

## Results

### Characteristics of the study participants

Table 1 shows the demographic and metabolic characteristics, including the WBC profile, of the 157 participants according to their HOMA2-IR value. There was no significant difference in sex between insulin-resistant and insulin-sensitive individuals. Compared to the insulin-sensitive participants, those who were insulin resistant had higher BMI, waist circumference, fasting plasma glucose and glycated hemoglobin, and lower HDL cholesterol but did not differ in total and LDL cholesterol.

**Table 1:** Demographic and metabolic characteristics of the study participants

	Insulin sensitive (*N*=92)	Insulin resistant (*N*=65)	*p*
Sex (#)	49	39	0.419
NGM (#)	62	22	0.000
IGM (#)	19	24	0.146
Type 2 diabetes (#)	11	19	0.04
Smoking status (#)	15	10	1
Lipid-lowering medication (#)	29	26	0.26
Glucose-lowering medication (#)	6	8	0.26
Age (years)	65.1 ± 7.1	65.6 ± 6.3	0.683
BMI (kg/m^2^)	27.2 ± 3.3	31.3 ± 4.5	< 0.001
Waist circumference (cm)	95 ± 8.9	108.6 ± 9.9	< 0.001
Fasting plasma glucose (mmol/L)	5.1 ± 0.6	5.7 ± 0.7	< 0.001
Glycated hemoglobin (mmol/mol)	42.1 ± 0.5	45.4 ± 0.6	0.006
Cholesterol (mg/L)	5.3 ± 1	5.2 ± 1.1	0.586
HDL (mg/L)	1.4 ± 0.3	1.2 ± 0.3	< 0.001
LDL (mg/L)	3.9 ± 1	3.5 ± 1	0.06

Data are mean and ± SD. *P* values were calculated with the Wilcoxon rank-sum test for the continuous variables and the chi-squared test for the categorical variables

*NGM* Normal glucose metabolism, *IGM* Impaired glucose metabolism

### Insulin-resistant individuals have a higher relative amount of whole blood monocytes compared to insulin-sensitive individuals

Compared to the insulin-sensitive participants, those who were insulin-resistant had a higher relative amount of monocytes (14 ± 7% vs 11 ± 4% FDR-*p*=0.029, Table 2); indicating more pronounced monocytosis [14, 15]. The relative amount of B cells, NK cells, CD4+ T cells, CD8+ T cells, neutrophils, and eosinophils did not differ between insulin-resistant and insulin-sensitive participants (Table 2).

**Table 2:** WBC profile for the study participants

	Insulin sensitive (*N*=92)	Insulin resistant (*N*=65)	*p*	FDR-*p*
B cells (%)	9 ± 3	8 ± 4	0.113	0.306
NK-cells (%)	11 ± 6	10 ± 6	0.299	0.418
CD4T cells (%)	28 ± 14	26 ± 9	0.131	0.306
CD8T cells (%)	10 ± 10	9 ± 9	0.697	0.814
Monocytes (%)	12 ± 4	14 ± 7	0.002	0.016
Neutrophils (%)	26 ± 16	29 ± 16	0.246	0.418
Eosinophils (%)	0 ± 0	0 ± 0	0.947	0.947

EpiDISH was used to estimate the WBC profile from the DNA methylation data. Data are median ± MAD (median absolute deviation). *P* values were calculated with the Wilcoxon rank-sum test. Multiplicity correction was performed by applying the Benjamini-Hochberg method to control the false discovery rate. The median eosinophil composition of 0% means very low abundance

Furthermore, we additionally adjusted the DNA methylation for three covariates, including smoking status, lipid- and glucose-lowering medication, to investigate their impact on WBC composition estimation. We showed that adjusting the DNA methylation data for smoking status, or lipid-/glucose-lowering medication does not affect the estimation of the WBC profile, as the relative amount of B cells, NK cells, CD4+ T cells, CD8+ T cells, neutrophils, and eosinophils did not differ between the study participants (Table 3).

**Table 3:** Differences in the WBC profile of the study participants, adjusted for smoking status, and lipid- and glucose-lowering medication

	Without adjustment (reference)	Smoking status	Lipid-lowering medication	Glucose-lowering medication	*p* Smoking status *vs* reference	*p* Lipid-lowering medication *vs* reference	*p* Glucose-lowering medication *vs* reference
B cells (%)	9 ± 4	9 ± 4	9 ± 4	9 ± 4	0.734	0.948	0.911
NK cells (%)	11 ± 6	11 ± 6	10 ± 6	11 ± 6	0.959	0.956	0.821
CD4T cells (%)	27 ± 12	26 ± 12	27 ± 12	27 ± 12	0.820	0.871	0.563
CD8T cells (%)	9 ± 10	10 ± 10	10 ± 10	10 ± 10	0.953	0.762	0.493
Monocytes (%)	12 ± 5	12 ± 5	12 ± 5	12 ± 5	0.814	0.956	0.736
Neutrophils (%)	27 ± 15	27 ± 15	27 ± 15	27 ± 15	0.959	0.827	0.885
Eosinophils (%)	0 ± 0	0 ± 0	0 ± 0	0 ± 0	0.753	0.810	0.781

EpiDISH was used to estimate the WBC profile from the DNA methylation data. Data are median ± MAD. *P* values were calculated with the Wilcoxon rank-sum test and the “without adjustment group” was used as a reference. The median eosinophil composition of 0% means very low abundance

### Effect of adjustment for WBC profile

**Fig. 1:**
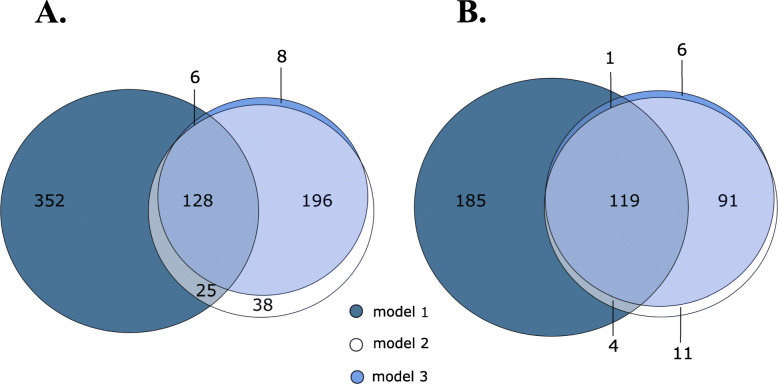
Venn diagram of the numbers of the significantly (nominal *p* < 0.05) upregulated (**A**) and downregulated (**B**) genes, comparing insulin-resistant to insulin-sensitive individuals. Three models were used: adjusted for sex, BMI, and age (model 1); additionally adjusted for the WBC profile (model 2); and additionally adjusted for lipid and glucose-lowering medication (model 3)

We performed RNA sequencing on whole blood to assess transcriptome differences between insulin-resistant and insulin-sensitive participants. The insulin-sensitive group was used as a reference and the analysis identified 511 significantly upregulated genes in model 1, 387 in model 2, and 338 in model 3 (Fig. 1A and Table S1). At the same time, we identified 309 genes significantly downregulated in model 1, 225 in model 2, and 217 in model 3 in the insulin-resistant *vs* the insulin-sensitive comparison (Fig. 1B and Table S2). Upon adjustment for multiple testing, none of these genes reached an individual FDR-*p* < 0.05. Figure 1 emphasizes the effect of adjusting for WBC profile compared to model 1, i.e. fewer differentially expressed genes. We anticipated that the adjustment for the WBC profile would reduce power in the models, since we introduced additional covariates; therefore, the reduced number of significant genes is not in itself unforeseen. Yet, the reduction in the number of differentially expressed genes was much stronger than would be expected from the added number of covariates and the genes that were no longer significant were mostly related inflammatory processes, which implies that these differences were due to differences in the WBC profile between insulin resistance and insulin-sensitive individuals (Figures S2 and S3).

**Fig. 2:**
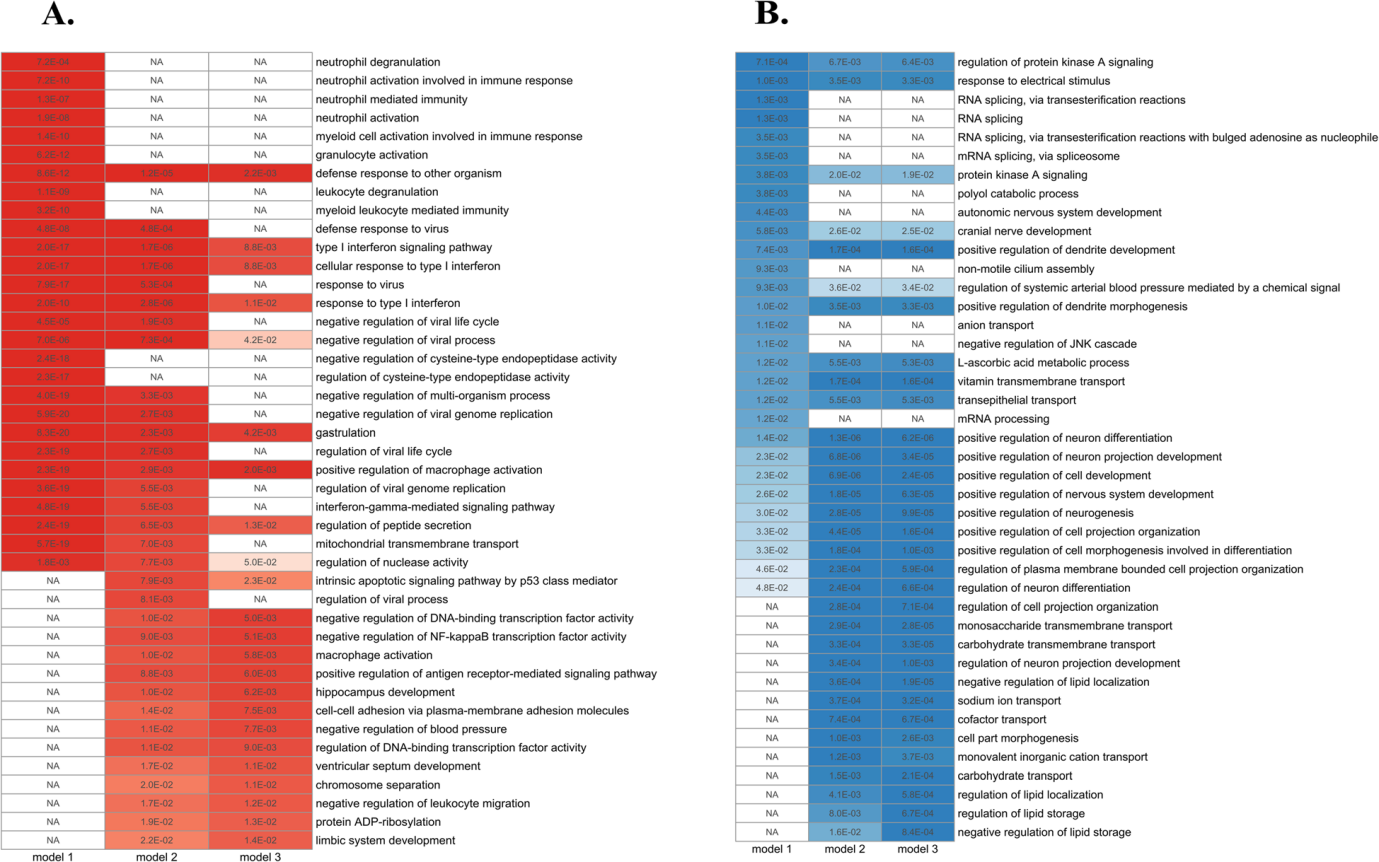
Heatmaps representing the top 20 GO biological processes for the comparison of insulin-resistant to insulin-sensitive individuals, for the upregulated (**A**) and downregulated (**B**) genes. Three models were used: adjusted for sex, BMI, and age (model 1); additionally adjusted for the WBC profile (model 2); and additionally adjusted for lipid and glucose-lowering medication (model 3). The GO biological processes were ranked based on their nominal *p* < 0.05. The color was based on the log_2_ fold change of the genes that were used as an input for the GO enrichment analysis; red gradients indicate upregulation and blue gradients downregulation. NA indicates that the process was not enriched

To illustrate this effect we show the top 20 biological processes (nominal *p* < 0.05) for each model. There was a clear effect on the significant biological processes when we additionally adjusted for WBC composition compared to model 1 (Fig. 2). More specifically, for the upregulated genes (Fig. 2A, first and middle column), several immune response processes (e.g., “neutrophil degranulation” or “neutrophil activation”) were not enriched in model 2, while for the downregulated genes (Fig. 2B, first and middle column) cellular processes (e.g., “RNA splicing” or “mRNA processing”) were not enriched in model 2. Finally, Figs. 1 and 2 reveal a great overlap in genes and biological processes between models 2 and 3, which showed that additional adjustment for lipid-modifying and glucose-lowering medication did not materially alter the results. The complete list of the GO biological processes is reported for all models in Tables S3–S4.

### Higher expression of interferon-stimulated genes in the insulin-resistant, independent of the WBC profile

**Fig. 3:**
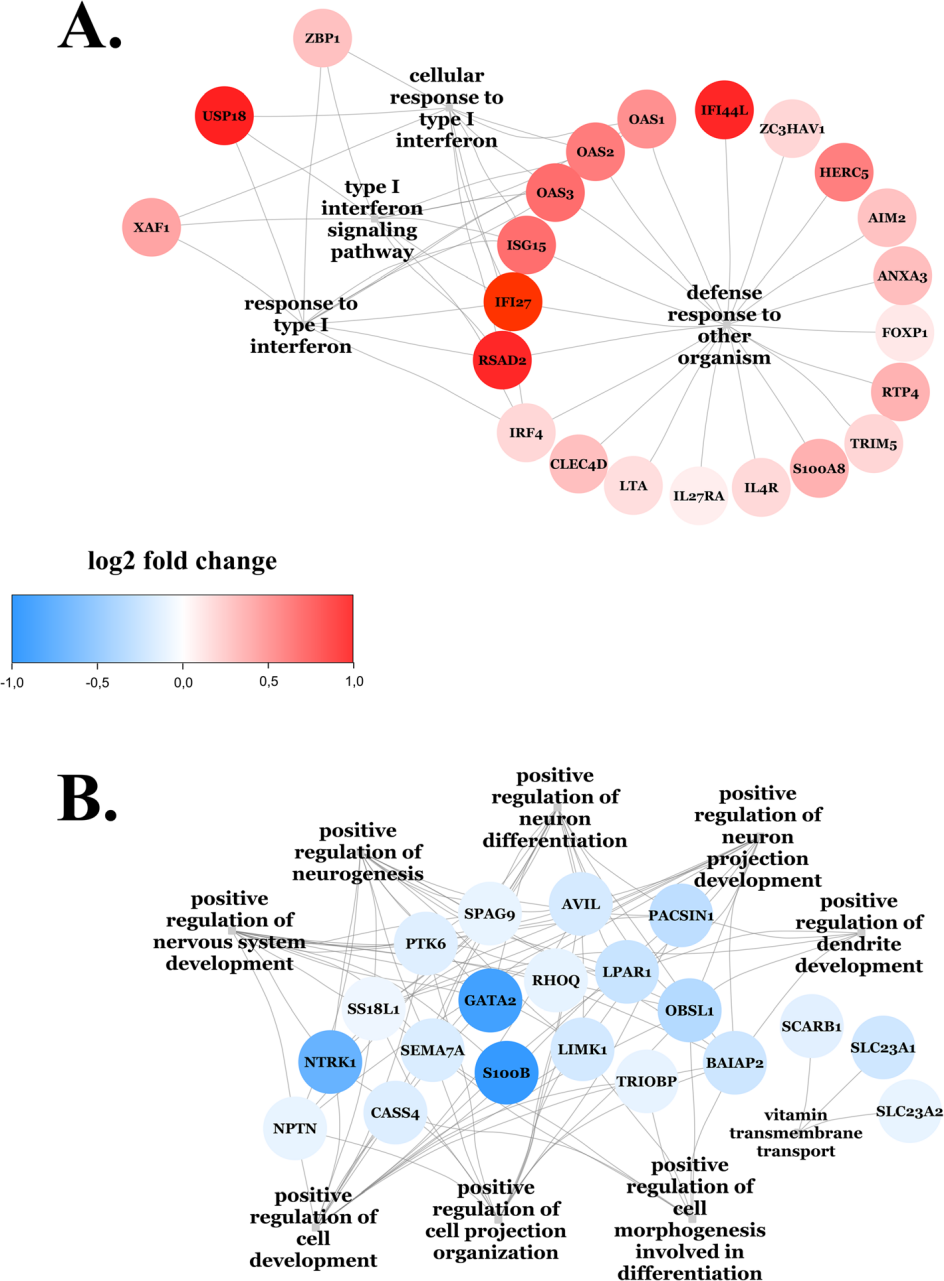
Two networks of the selected GO biological processes for the comparison of insulin-resistant to insulin-sensitive individuals after adjustment for WBC profile (model 2) for the upregulated (**A**) and downregulated (**B**) genes. The gene expression log2 fold changes are visualized on the nodes of the network. Genes are visualized as circles; the colors are based on their log2 fold change, and hence, the gradients of red indicate upregulation and blue indicate downregulation

The GO enrichment analysis for the upregulated genes, after adjusting for the WBC profile (model 2) resulted in four significant (FDR-*p*< 0.05) biological processes. The four significant biological processes were combined in a network (Fig. 3A). In total, twenty-three genes within these significant biological processes were unique and upregulated in the insulin-resistant group. More specifically, they include interferon-stimulated genes (ISGs), where interferons induce the expression of those genes, e.g., *ISG15*, *OAS1*, *OAS2*, *OAS3*, *RASD2*, *USP18*, *IFI27*, *IFI44L*, *HERC5*, *ZC3HAV1*, *FOXP1*, *ANXA3*, *RTP4*, *AIM2*, *TRIM5*, *ZBP1*, and *XAF1*. Other genes upregulated in the insulin-resistant individuals were involved in different innate immune response processes, e.g., *IRF4*, *LTA*, *S100A8*, *IL4R*, *IL27RA*, and *CLEC4D*. Collectively, these data show an interferon-related signature characterizing the whole blood transcriptome profile of the insulin-resistant individuals.

### Lower expression of genes involved in cellular differentiation and remodeling of actin cytoskeleton in insulin resistance, independent of the WBC profile

The GO enrichment analysis for the downregulated genes, after adjusting for the WBC profile resulted in nine significant (FDR-*p* < 0.05) biological processes. These nine significant biological processes were combined in a network (Fig. 3B). In total, twenty genes were unique and downregulated in the insulin-resistant group. More specifically, they include (i) genes involved in cellular differentiation and adhesion, e.g., *GATA2*, *S100B*, *PTK6*, *SEMA7A*, SPAG9, *NTRK1*, *NPTN*, *SS18L1*, and *CASS4*. Furthermore, we identified (ii) genes involved in the reorganization of the actin cytoskeleton, e.g., *LPAR1*, *RHOQ*, *LIMK1*, *TRIOBP*, *AVIL*, *PACSIN1*, *BAIAP2*, and *OBSL1*, along with genes involved in (iii) vitamin C transport, e.g., *SLC23A1* and *SLC23A2* and (iv) a gene that promotes hepatic uptake of cholesterol from HDL (*SCARB1*). Collectively, these data show the downregulation of cellular differentiation and actin cytoskeleton remodeling in the insulin-resistant group.

### Analysis to test the effect of lipid-/glucose-lowering medication

Exclusion of those who use lipid-modifying and/or glucose-lowering medication did not materially alter the results (Tables S5, S6, S7 and S8).

## Discussion

In this paper, we present an approach that employs DNA methylation data to investigate the whole blood transcriptome of insulin-resistant and insulin-sensitive individuals from the CODAM study, independent of the WBC profile. We used the whole blood transcriptome as a means that reflects the whole blood environment along with insulin resistance-related organ-specific changes. We showed the relative amount of monocytes to be significantly greater in the insulin-resistant participants. Furthermore, we demonstrated a distinct whole blood transcriptome profile in insulin resistance, independent of the WBC profile. More specifically, we showed a higher expression of ISGs and a lower expression of genes related to cell differentiation and actin remodeling characterizing individuals with insulin resistance.

The higher percentage of monocytes in the insulin-resistant participants is in line with previous findings [14, 15]. More specifically, De Matos et al. found that insulin-resistant, but not insulin-sensitive, obese individuals, had an increased percentage of CD14+ CD16+ monocytes compared to healthy control [14]. In addition, Poitou et al. described a higher percentage of CD16+ monocytes in obese insulin-resistant individuals [15]. Hyperglycemia per se may also promote myelopoiesis, which in turn results in increased production and infiltration of monocytes into the atherosclerotic plaques [16, 17]. Interestingly, both insulin resistance and hyperglycemia are risk factors for atherosclerosis [18]. Upon entry into a tissue, monocytes differentiate into macrophages and dendritic cells and exert effects on the tissue homeostasis. For example, inhibition of colonic macrophage recruitment in the gut promotes insulin sensitivity [19], and accumulation of macrophages in adipose tissue is associated with insulin resistance [20]. Thus, causal relationship between monocytosis on the one hand and insulin resistance and hyperglycemia on the other may comprise a vicious cycle in which the order of events is unclear. Hence, we do not know whether the higher percentage of monocytes we observe in the obese/overweight insulin-resistant individuals is a cause or consequence of insulin resistance.

Furthermore, the observed overrepresentation of monocytes suggests a relative underrepresentation of other cell types. Since each circulating WBC subtype is characterized by the expression of a certain set of genes, we anticipated that the differences in the WBC profile between insulin-resistant and insulin-sensitive participants would drive overall whole blood gene expression. Hence, by adjusting for differences in the WBC profile we remove this confounding effect. The effect of inter-person differences in a cellular profile and its contribution to bulk tissue gene expression has been previously reported [21–25]. Briefly, biological tissue samples are characterized by heterogeneous and varying cellular composition. Particularly if a researcher is primarily interested in the identification of changes in expression of genes across conditions, concomitant changes in cell type composition can hamper data interpretation, as detecting differentially expressed genes is strongly affected by the sample variation in cell-type frequencies. In particular, Shen-Orr and Gaujoux concisely summarized and proposed several ways to adjust for cellular profile changes, e.g., to include them as covariates in a regression model [23]. In line with our results, Qiao et al. reported different enriched processes in human blood when adjusting for cellular profile changes [24]. Additionally, and similarly to our results, Zhuang et al. reported less differentially expressed genes when adjusting for the cellular profile in hippocampal gene expression in mouse models of Alzheimer’s disease [25].

As we show in Fig. 1 and in Figures S2 and S3, before adjustment for the WBC profile, immunological pathways were overrepresented in the GO analysis. This became less apparent after adjustment, indicating that these immunological pathways were actually a reflection of the WBC composition. Furthermore, for the upregulated genes (Figure S2, first and middle column), several genes usually involved in immune response processes (e.g., *IFIT1*, *IFI6*, *IRF7*, *IRF9*, *IL1RN*) were not differentially expressed in model 2. On the same note, for the downregulated genes (Figure S3, first and middle column), several genes usually involved in intracellular processes (e.g., *PDZK1*, *UST*, *PTGDR*, *CHCHD7*) were not differentially expressed in model 2. Interestingly, several of the up- and downregulated genes became differentially expressed after adjusting for the WBC profile. Taken together, adjustment for WBC profile in the whole blood, as we did in our current differential gene expression analysis, adds a layer of information that would otherwise remain elusive while substantially improving the biological interpretation of the data.

We observed higher expression of the S100 Calcium Binding Protein A8 (*S100A8*) gene in the insulin-resistant individuals, independent of the WBC profile. Increased gene expression of *S100A8* in whole blood has been described in individuals with the metabolic syndrome [8] and obese individuals [26] and associated with ROS generation [8, 26]. Furthermore, studies in humans and mice have reported increased expression of *S100A8* and *S100A9* in adipose tissue of obese and diabetic subjects [27, 28]. Interestingly, in our analysis, the *S100A9* was not significant after adjusting for the WBC profile. Nevertheless, calprotectin advances ROS generation binds to the toll-like receptor 4 and receptor for advanced glycation and products (RAGE), important signaling pathways involved in the pathogenesis of atherosclerosis [26]. Yamaoka et al. hypothesized that the *S100A8* (as well as *S100A9* and *S100A12*) gene might be involved in the development of atherosclerosis and type 2 diabetes [26]. In our analysis, the higher expression of the *S100A8* gene in insulin resistance is independent of the WBC profile, but the WBC profile activity and the S100A8/A9-RAGE pathway may be of importance in the etiology of atherosclerosis.

Furthermore, we observed that, independent of the WBC profile, the expression of ISGs in the whole blood was higher in insulin-resistant as compared to insulin-sensitive individuals. A positive association between BMI of obese subjects and the increased expression of type I interferon ISGs has been previously reported in human PBMCs [29]. Furthermore, van der Pouw Kraan et al. described an increase in type I interferon ISGs expression in type 2 diabetes subjects [30]. ISGs constitute a group of genes that are upregulated in response to interferon, indicating increased inflammation due to an innate immune response [31]. Obese individuals are prone to infections and have a worsened immune response to viral and bacterial infections [32]. Interestingly, it has been previously shown that viral infections can increase leukocyte interferon production and may cause systemic insulin resistance [33] or muscle insulin resistance [34]. Obesity is also characterized by low-grade inflammation and secretion of pro-inflammatory cytokines into circulation, which may advance insulin resistance [1]. Hence, this interferon-related signature indicates increased systemic inflammation possibly due to an innate immune response and whole-body insulin resistance, which can be a cause or consequence of insulin resistance.

Independent of the WBC profile, we additionally observed a lower expression of genes involved in cellular differentiation and reorganization of the actin cytoskeleton, in the whole blood of insulin-resistant individuals. Cellular differentiation is regulated through activation and repression of transcription factors and is associated with changes in cell shape, mainly determined by the dynamics of the actin cytoskeleton. Interestingly, the transcription factor *GATA2* and the calcium-binding protein *S100B* were among the most downregulated genes in the insulin-resistant individuals. Tangen et al. reported *GATA2* downregulated in whole blood gene expression profiles in individuals with the metabolic syndrome [8]. Additionally, Okitsu et al. reported that interferon-gamma decreased *GATA2* expression in TBR343 cells (pre-adipocyte stromal cell line), suggesting that interferon-gamma may influence phenotypic changes in both hematopoietic and mesenchymal cells by suppression of *GATA2* [35].

Yamaoka et al. showed a positive correlation between the whole blood *S100A8* gene and visceral fat adiposity, which was strongly associated with measures of insulin resistance and inflammation [26]. Ghosh et al. reported that induction of type I interferon in visceral adipose tissue contributes to low-grade inflammation by stimulating polarization of pro-inflammatory macrophages, therefore advancing adipose and systemic insulin resistance in obese individuals [36]. Lee et al*.* reported increased expression of ISGs in the adipocytes of obese subjects, suggesting a reaction to interferon secreted by lymphocytes [37]. Previous studies have hypothesized that altered gene expression in specific organs may be reflected in whole blood or blood cells [10, 30]. Hence, we think that the systemic inflammation in combination with the downregulation of cellular differentiation and remodeling of actin cytoskeleton we observe in the whole blood of the insulin-resistant individuals may reflect obesity and/or insulin resistance-related organ dysfunction (e.g., adipose tissue or gut) in the insulin-resistant individuals.

The main strength of this study is the use of the computational algorithm EpiDISH [13] and the DNA methylation data to infer the WBC profile, as an alternative approach to transcriptomics inferred WBC profile. Using the DNA methylation data instead of the transcriptomics data from CODAM to infer the WBC profile enabled us to perform a differential gene expression analysis without the risk of introducing bias. Also, we adjusted for differences in WBC profile because we anticipated that the difference in WBC profile between insulin-resistant and insulin-sensitive individuals would drive overall whole blood gene expression. Indeed, the GO analysis before adjustment was overrepresented by immunological pathways, which became less apparent after adjustment. Finally, compared to flow-cytometry, computational algorithms (e.g., EpiDISH, CIBERSORT) are inexpensive, readily available, and extensively validated against flow-cytometric (FACS) estimates [11, 13]. On that note, EpiDISH is able to handle whole blood samples; estimates for the relative proportions of PBMC subtypes (lymphocytes (T cells, B cells, NK cells) and monocytes) within the PBMC pool are consistent when using whole blood or PBMC only samples [13].

A limitation of this study is that we do not have absolute numbers for the WBC subtypes. Absolute numbers of cell type amounts in a tissue are important for prognosis and diagnosis in clinical applications [13]. In clinical applications, the WHO in HIV treatment guidelines accepts relative numbers, but in medical practice, the decision for treatment depends on absolute numbers (cell counting per volume) [38]. Therefore, it would be interesting for future studies to investigate the differences in adjustment for relative or absolute differences in WBC profile on blood transcriptome analysis and subsequent biological interpretation in the context of obesity/insulin resistance.

Taken together, if DNA methylation and RNA sequencing data are available, we would recommend our proposed approach to other researchers, as it enables a differential gene expression analysis without the risk of introducing bias caused by differences in cell type composition between individual samples, thereby enabling a more comprehensive biological interpretation. To our knowledge, this cross-sectional analysis is the first to show that an interferon-related signature characterizes the whole blood transcriptome profile of overweight/obese insulin-resistant individuals, independent of WBC profile, which can be a cause or consequence of insulin resistance. In addition, we show higher expression of the *S100A8* gene in insulin resistance independent of the WBC profile. Recently the beneficial role of the hematopoietic-restricted deletion of *S100A8* and *S100A9* in preventing monocytosis has been reported [17], while Yamaoka et al., hypothesized that the *S100A8* gene might be involved in the development of atherosclerosis [26]; therefore, we speculate that the higher expression of the *S100A8* gene in insulin resistance independent of the WBC profile, may be of importance in the etiology of atherosclerosis. Our findings of the differential whole blood transcriptome in insulin resistance may provide targets and biomarkers for more personalized risk classification in the prevention and treatment of cardiometabolic disease.

## Conclusions

In conclusion, we have presented an approach to investigate the whole blood transcriptome of insulin-resistant individuals, independent of their DNA methylation-derived WBC profile. We reported a 3% higher relative amount of monocytes in the insulin-resistant individuals. We anticipated that the differences in WBC profile between insulin-resistant and insulin-sensitive individuals would affect overall whole blood gene expression, therefore we adjusted for the WBC profile. We observed a higher expression of ISGs in the whole blood in insulin-resistant individuals, independent of the WBC profile. This interferon-related signature indicates increased systemic inflammation possibly due to an innate immune response and whole-body insulin resistance. We additionally observed a lower expression of genes involved in cellular differentiation and reorganization of the actin cytoskeleton, in the whole blood of insulin-resistant individuals, independent of WBC profile. Altered gene expression in specific organs may be reflected in whole blood; hence, we think that our results may reflect obesity and/or insulin resistance-related organ dysfunction in the insulin-resistant individuals.

## Methods

### Subject characteristics

The CODAM study is a prospective observational cohort that includes participants with an elevated risk of T2DM and cardiovascular disease. Participants were selected from a large population-based cohort as previously described [39]. Briefly, inclusion criteria were Caucasian descent and age > 40 years and, in addition, at least one of the following: BMI > 25 kg m^2^; a family history of T2DM; a history of gestational diabetes and/or glucosuria; and use of anti-hypertensive medication. Combinations of these separate inclusion criteria were also allowed. The participants were extensively characterized with regard to their metabolic, cardiovascular, and lifestyle profiles during two visits to the University’s research unit. Data were collected for 574 participants at baseline and for 495 participants at follow-up of 7 years. Whole blood DNA methylation and transcriptome data were generated in the follow-up samples of participants who had been free of cardiovascular disease and type 2 diabetes at baseline. A prerequisite for our participants was to have HOMA2-IR values, DNA methylation, and RNA sequencing data. Individuals without these data were not included in our analysis. Hence, the final dataset included 157 participants (Figure S1). The medical ethics committee of the Maastricht University approved this study. All participants gave written informed consent.

### Assessment of insulin resistance by Homeostasis Model Assessment

Insulin resistance was estimated according to the HOMA using the HOMA2 calculator (http://www.dtu.ox.ac.uk) [40]. A HOMA2-IR cut-off value of 1.7 was chosen based on the 75th percentile [41] of the participants with normal glucose metabolism because they provide the best approximation of the normal values of the CODAM study population; thus, participants with HOMA2-IR above or equal to 1.7 were defined as insulin resistant, whereas those with HOMA2-IR below 1.7 were defined as insulin sensitive.

## Statistical analyses

### Participant characteristics

We assessed the phenotypic differences between the insulin-resistant and insulin-sensitive participants. Because the majority of continuous variables were not normally distributed, the median and median absolute deviation (MAD) were shown. In addition, for the same reason, a Wilcoxon rank-sum test for the continuous variables and a chi-squared test for the categorical variables was used. Multiplicity correction was performed on the *p* values by applying the Benjamini-Hochberg method to control the false discovery rate (FDR). The threshold for statistical significance was set at *p* < 0.05 for nominal and FDR *p* values.

### Estimating WBC blood profiles using DNA methylation data

#### Genome-wide DNA methylation analysis

A detailed description regarding the generation and processing of the DNA methylation data can be found elsewhere [42]. Briefly, DNA was obtained from the antecubital vein and the buffy coat (representing the white blood cell fraction of the whole blood sample) was collected for DNA isolation. The Zymo EZ DNA methylation kit was used to bisulfite-convert 500 ng of genomic DNA, and 4 μl of bisulfite-converted DNA was measured on the Illumina Human Methylation 450 array according to the manufacturer’s protocol. The methylation level was quantified in beta-values [43]. The beta-value, ranging from 0 to 1, is the ratio of the methylated probe intensity and the overall intensity (sum of methylated and unmethylated probe intensities), representing the fraction of methylated CpGs at a given single CpG locus. The final dataset consisted of 485,512 methylation probes measured in 157 samples.

The WBC profile in whole blood was determined through the Bioconductor package EpiDISH (v2.2.2), a computational algorithm that estimates tissue cell fractions from DNA methylation data [13]. EpiDISH is an extension of the CIBERSORT algorithm [11], which is a general framework that can be applied to diverse cell phenotypes and genomic data types.

Briefly, as input, EpiDISH requires a “signature matrix” comprised of genomic loci that have distinct DNA methylation patterns between the cell types of interest. Once a suitable knowledge base is created and validated, EpiDISH can be applied to characterize cell type proportions in bulk tissue DNA methylation profiles. The DNA methylation beta values served as input for the CIBERSORT algorithm [11] to calculate the amounts of a priori known cell subtypes from DNA methylation in blood. EpiDISH estimates the WBC profile from the relative amount of seven WBC subtypes, namely B cells, NK cells, CD4+T cells, CD8+T cells, monocytes, neutrophils, and eosinophils.

We additionally adjusted the DNA methylation for three covariates, including smoking status, lipid- and glucose-lowering medication, to investigate their impact on WBC composition estimation. In their review, Li et al. mention several epigenome-wide association studies that found associations between smoking status and CpG methylation levels at specific genomic loci [44]. Furthermore, there is evidence of DNA methylation as a potential mechanism, by which lipid-/glucose-lowering medications use, e.g., statins or metformin, can lead to adverse metabolic alterations and subsequently type 2 diabetes [45, 46]. If these loci are also used as cell type-specific loci to estimate cell type composition, this may result in confounded composition estimates for smokers or individuals that take lipid-/glucose-lowering medication. Hence, three linear models were implemented (i) adjusted for smoking status (current smoker = 1, non-smoker = 0), (ii) adjusted for lipid-lowering medication (on lipid-lowering medication = 1, without lipid-lowering medication = 0), and (iii) adjusted for glucose-lowering medication (on glucose-lowering medication = 1, without glucose-lowering medication = 0). The DNA methylation beta values were transformed to *M*-values with the function *beta2m* [47]. The beta coefficient of the covariates of each independent model was subtracted from the *M*-values. Finally, the M-values were transformed again to beta values with the function *m2beta* [47], and the WBC profile was estimated anew.

### Transcriptome analysis

Briefly, whole blood was collected into PAX gene Blood RNA tubes according to the manufacturer’s instruction and stored at -80 until use. Total RNA was extracted from the whole blood as previously described [48] and the sequencing reads were mapped to human genome (HG19). Only reads with both ends mapping onto a single gene were considered. The final dataset consisted of 46,628 protein coding and non-coding genes measured in 157 samples.

### Differential gene expression analysis

After preprocessing, we performed a differential gene expression analysis on RNA-sequencing data for 46,628 genes and 157 samples using the Bioconductor package edgeR (v3.28.1) [49]. Before fitting a linear model*,* firstly pre-filtering of low count genes was applied, where genes that have CPM values above 0.5 in at least two libraries are kept [50]; hence, 23,787 genes remained. Secondly, based on the FPKM values that were calculated, genes with at least 1 FPKM in 5% of the samples were kept [51]. Eventually, in the final dataset 17,658 genes remained measured in 157 samples. The differential expression analysis implements a negative binomial generalized linear model with the insulin-resistant and insulin-sensitive groups; the insulin-sensitive group was used as a reference. Three models were used: (i) adjusted for sex, BMI, and age (henceforth referred to as model 1); (ii) additionally adjusted for WBC profile (model 2); and (iii) additionally adjusted for differences in lipid and glucose-lowering medication (model 3). All significantly differentially expressed genes (nominal *p*< 0.05) were divided into up- (nominal *p*< 0.05 and log2 fold change > 0) and downregulated (nominal *p*< 0.05 and log2 fold change < 0) genes.

### Gene Ontology analysis

Gene Ontology (GO) enrichment analysis was performed for each of the three models separately using the Bioconductor package clusterProfiler (v3.16.1) [52]. The ontology biological process was used. All significantly expressed genes were included, divided into up- and downregulated genes to provide direction for the involved biological processes [23].

### Network analysis

To facilitate interpretation, the significant (based on an individual FDR-*p* < 0.05) GO biological processes were imported and visualized as networks were in Cytoscape (v3.8.0) [53] with the function *createNetworkFromIgraph* from the Bioconductor package RCy3 (v3.11) [54]. The gene expression data were visualized on the nodes of the networks.

### Analysis to test the effect of lipid-/glucose-lowering medication

The differential gene expression analysis was repeated excluding the participants that were taking lipid- and/or glucose-lowering medication. This reduced dataset consisted of 17 651 genes in 99 samples. Two linear models were used with the insulin-resistant and insulin-sensitive groups (the insulin-sensitive group was used as a reference): (i) adjusted for sex, BMI, and age and (ii) additionally adjusted for the WBC profile. GO analysis was performed as described in the previous section.

## Supplementary Information

**Additional file 1: Figure S1-S3.**

**Additional file 2: Table S1.**

**Additional file 3: Table S2.**

**Additional file 4: Table S3.**

**Additional file 5: Table S4.**

**Additional file 6: Table S5.**

**Additional file 7: Table S6.**

**Additional file 8: Table S7.**

**Additional file 9: Table S8.**

## Data Availability

DNA methylation and RNA expression data are available from the corresponding author for any interested researcher who meets the criteria for access to confidential data and upon reasonable request. Other data are unsuitable for public deposition due to ethical restrictions and privacy of participant data.
