# Supplementary material for: An interferon-related signature characterizes the whole blood transcriptome profile of insulin-resistant individuals—the CODAM study
Source: Genes Nutr. 2021 Dec 9;16:22. doi: 10.1186/s12263-021-00702-7 (PMC8903498; doi:10.1186/s12263-021-00702-7)
Supplement: Supplementary file 1 — Additional file 1: Figure S1-S3. [file 12263_2021_702_MOESM1_ESM.pdf]

An interferon related signature characterizes the whole blood transcriptome profile of insulin resistant individuals – The CODAM study

Marianthi Kalafati[1], Martina Kutmon[2,4], Chris T. Evelo[2,4], Carla J.H. van der Kallen[3], Casper G. Schalkwijk[3], Coen D.A Stehouwer[3], BIOS Consortium, Ellen E. Blaak[1], Marleen M.J. van Greevenbroek[3,*] and, Michiel Adriaens [4,*]

*These authors contributed equally to this work

[1]Department of Human Biology, School of Nutrition and Translational Research in Metabolism (NUTRIM), Maastricht University, Maastricht, the Netherlands

[2]Department of Bioinformatics – BiGCaT, School of Nutrition and Translational Research in Metabolism (NUTRIM), Maastricht University, Maastricht, the Netherlands

[3]Department of Internal Medicine, School for Cardiovascular Diseases (CARIM), Maastricht University, Maastricht, the Netherlands

[4]Maastricht Centre for Systems Biology (MaCSBio), Maastricht University, Maastricht, the Netherlands

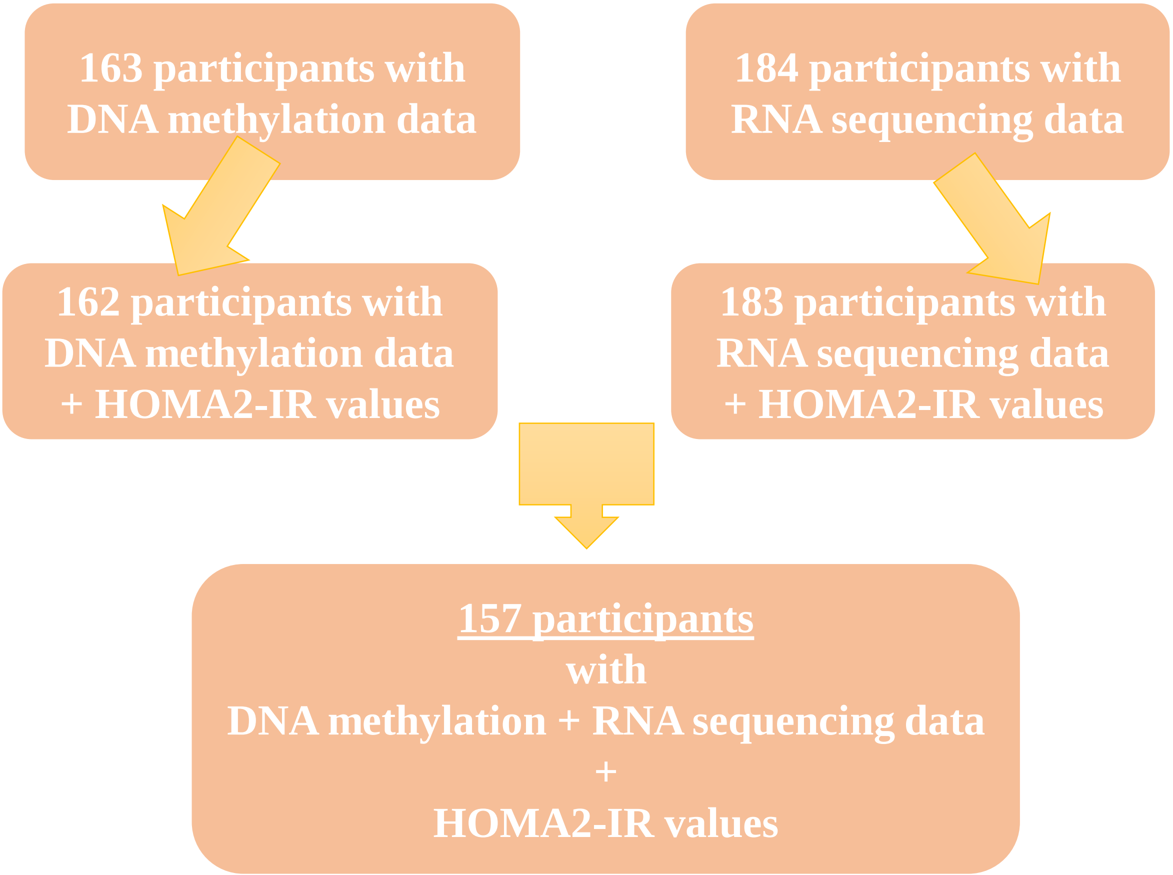

Figure S1. Selection flow of the CODAM study participants. In our study 157 participants were included with DNA methylation and RNA sequencing data and HOMA2-IR values. The CODAM study has DNA methylation data for 163 participants and RNA sequencing data for 184 participants. Of those, 162 participants with DNA methylation data have HOMA2-IR values and 183 with RNA sequencing data have HOMA2-IR values. In order to perform the differential gene expression analysis and adjust for differences in WBC profile our participants had to have HOMA2-IR values, DNA methylation and RNA sequencing data, therefore the final dataset comprised of 157 participants.

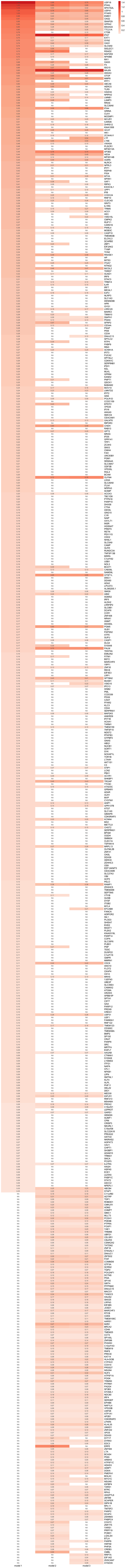

model 1
model 2
model 3
1.4
1.2
1
0.8
0.6
0.4
0.2

Figure S2. Heatmap representing the upregulated genes (nominal $p<0.05$) for the comparison of insulin resistant to insulin sensitive individuals. Three models were used: adjusted for sex, BMI and age (model 1), additionally adjusted for the WBC profile (model 2) and additionally adjusted for lipid and glucose lowering medication (model 3). The genes were ranked based their nominal $p<0.05$ in regards to model 1 (first column). The color was based on the $\log_2$ fold change of the genes; red gradients indicate upregulation. NA indicates that the genes were not differentially expressed.

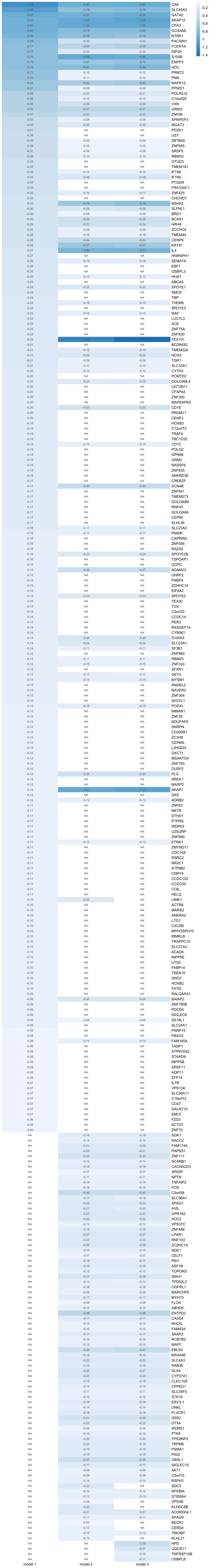
-0.2
-0.4
-0.6
-0.8
-1
-1.2
-1.4
CA8
SLC45A3
GATA2
AKAP12
CPA3
GCSAML
NTRK1
PACSIN1
FCER1A
DIP2A
S100B
ENPP3
HDC
PRMT2
PNN
MAPK12
PPWD1
POLR2J3
C14orf28
VXN
GRIK5
ZNF66
XPNPEP3
MGAT3
PDZK1
UST
ZBTB49
ZNF655
SRSF5
RBM33
OTUD3
TMEM181
IFT88
IFT80
PTGDR
FRA10AC1
ZNF425
CHCHD7
MS4A2
SLFNL1
BRD1
BCAS1
HRH4
ZCCHC4
TMEM44
CENPK
KRT81
IL4
HNRNPH1
SEMA7A
EBF1
OSBPL3
HHAT
ABCA5
SPDYE1
SMC6
TBP
THEM5
SPDYE3
MAF
LUC7L3
ACE
ZNF75A
ZNF830
TEX101
BCDIN3D
TMEM220
HCN3
TGIF1
SLC33A1
CYTH3
PCMTD2
GOLGA6L4
UGT2B11
CFAP44
ZNF300
MAPKAPK5
CD1E
PRDM11
CENPJ
HOXB3
C12orf73
TRAF4
TBC1D32
CD1C
POLG2
GPR68
GRM2
RASSF6
ZNF835
ANKRD36
CREBZF
SCN4B
ZNF841
TMEM273
GOLGA8B
RNF43
GOLGA8A
CEP95
KLHL36
SLC23A2
PNISR
CAPRIN2
ZNF506
RAD52
SPDYE2B
TSPOAP1
GOPC
ADAM23
UHRF2
FNBP4
ZDHHC14
EIF5A2
SPDYE2
TEX30
TOX
C3orf33
CCDC14
PER3
RASGEF1A
CYB561
S100A5
SLC23A1
SF3B1
ZNF880
RBM25
ZNF333
SFXN1
GET4
MYSM1
ANGEL2
RAVER2
ZNF304
SH3YL1
PODXL
MINAR1
ZNF26
NDUFAF6
SNRPN
CD200R1
ZC3H8
CEP85L
L2HGDH
OXCT1
MSANTD4
ZNF793
DUSP2
PLG
SREK1
MASP2
AKAP1
GK5
ADRB2
ZNF83
NKTR
DTHD1
PTPRS
WDR53
U2SURP
ZNF680
ETNK1
ZMYND11
CDC14A
RSRC2
NR2C1
ATP8B2
CNPY4
CCDC122
CCDC50
COIL
HELQ
LIMK1
ACTR8
MARS2
ANKRA2
LTO1
CXCR6
MPHOSPH10
RIMKLB
TRAPPC10
SLC27A2
ACAD8
INPP5E
UTS2
FKBP14
TSEN15
GNG7
HOXB2
FKTN
RALGAPA1
BAIAP2
ZNF780B
PDCD4
SIGLEC6
SS18L1
SLC24A1
PARP15
FBXO3
FAM185A
TASP1
ATP6V0A2
STARD9
INPP5B
SRSF11
AQP11
ZFP14
IL7R
VPS13A
SLC26A11
C19orf12
CD47
GALNT12
EML5
FZD3
KCTD7
ZNF70
SGK1
NACC2
FAM174A
PAPSS1
ZNF117
SCARB1
CACNA2D3
SPIDR
NPTN
TNFAIP2
FOS
C5orf58
SLC36A1
SPAG1
AVIL
GPR162
AOC2
VPS37C
ZNF486
LPAR1
RNF103
ZC2HC1A
NDE1
CELF1
PID1
ASF1B
TOPORS
SIAH1
TPD52L2
OGFRL1
MARCHF6
MYH10
FLCN
ABHD5
ENTPD2
CASS4
RHOQ
FAM43A
SKAP2
RCBTB2
MAFF
EBLN2
MS4A4E
SLC4A3
RAB36
DLX6
CYP27A1
CLEC10A
CPPED1
SLC35F5
STK19
ERV3-1
UNKL
FLVCR1
G0S2
DTX4
WDR91
PTK6
TP53INP2
TRPM6
PSMA1
PIGZ
OBSL1
SIGLEC15
AKT1
C5orf15
RSPH3
SDC3
NFKBIA
ST8SIA4
VPS4B
KLHDC8B
AC090004.1
SPAG9
BECN1
CERS4
TRIOBP
KLHL21
HPD
UQCR11
TNFRSF10B
OSBPL8
model 1
model 2
model 3

Figure S3. Heatmap representing the downregulated genes (nominal $p<0.05$) for the comparison of insulin resistant to insulin sensitive individuals. Three models were used: adjusted for sex, BMI and age (model 1), additionally adjusted for the WBC profile (model 2) and additionally adjusted for lipid and glucose lowering medication (model 3). The genes were ranked based their nominal $p<0.05$ in regards to model 1 (first column). The color was based on the log_2 fold change of the genes; blue gradients downregulation. NA indicates that the genes were not differentially expressed.
